# The Establishment and Practice of Pharmacy Care Service Based on Internet Social Media: Telemedicine in Response to the COVID-19 Pandemic

**DOI:** 10.3389/fphar.2021.707442

**Published:** 2021-10-01

**Authors:** Huibo Li, Siqian Zheng, Da Li, Dechun Jiang, Fang Liu, Wei Guo, Zhenying Zhao, Yanfei Zhou, Jingting Liu, Rongsheng Zhao

**Affiliations:** ^1^ Department of Pharmacy, Peking University Third Hospital, Beijing, China; ^2^ Institute for Drug Evaluation, Peking University Health Science Center, Beijing, China; ^3^ Department of Pharmacy, Raffles Hospital Beijing, Beijing, China; ^4^ Beijing Pharmacists Association, Beijing, China; ^5^ Department of Pharmacy, Beijing Shijitan Hospital, Capital Medical University, Beijing, China; ^6^ The National Clinical Research Center for Mental Disorders & Beijing Key Laboratory of Mental Disorders, Department of Pharmacy, Beijing An’ding Hospital, Capital Medical University, Beijing, China; ^7^ Advanced Innovation Center for Human Brain Protection, Capital Medical University, Beijing, China; ^8^ Department of Pharmacy, Tianjin Union Medical Center, Tianjin, China

**Keywords:** COVID-19, medication therapy management (MTM), remote pharmacy service, innovative service model, telepharmacy

## Abstract

**Objective**: For patients with chronic diseases requiring long-term use of medications who are quarantined at home, the management of medication therapy during the COVID-19 pandemic is a problem that pharmacists urgently need to discuss and solve. The study aims to establish and launch a telepharmacy framework to implement pharmaceutical care during the COVID-19 pandemic.

**Methods**: To establish a remote pharmacy service model based on a medication consultation service platform under the official account of the “Beijing Pharmacists Association” on the social software WeChat app, obtain the medication consultation records from February 28 to April 27, 2020, during the worst period of the epidemic in China, and to perform a statistical analysis of the information about the patients seeking consultation, consultation process, content and follow-up results.

**Results**: The medication consultation service system and telepharmacy service model based on social software were established in February 2020. The “Cloud Pharmacy Care” platform had 1,432 views and 66 followers and completed 39 counseling cases in 2 months. Counseling was available for patients of all ages. Of the 39 cases, 82.05% of patients were young and middle-aged. During the COVID-19 pandemic, the long-term medication usage problems of patients with chronic disease were effectively addressed using “Cloud Pharmacy Care”. In the consultation, 35 cases (89.7%) were related to the use of medicines or health products, and 4 cases (10.3%) involved disease state management and the use of supplements. The top five drug-related issues included the selection of medications, the dosage and usage of drugs, medications for special populations, medication therapy management of chronic diseases, and adverse drug reactions. All consultations were completed within 4 h, with a positive review rate of 97.4%.

**Conclusion**: During the COVID-19 pandemic, a remote pharmacy service “Cloud Pharmacy Care” based on the social software WeChat app was quickly constructed and applied to solve the medication-related problems of patients and the public during home quarantining. The significance of the study lies in the timely and interactive consultation model helps to carry out medication therapy management for chronically ill patients and improves patients’ medication compliance, improves medical quality, and plays a positive role in promoting the popularization of safe medication knowledge.

## Introduction

In December 2019, a cluster of cases of acute respiratory illness caused by respiratory syndrome coronavirus 2 (SARS-CoV-2) were detected and then spread rapidly worldwide (Chen et al., 2020; Li et al., 2020; Zhu et al., 2020). The World Health Organization (WHO) declared that the 2019 coronavirus disease (COVID-19) outbreak was the sixth Public Health Emergency of International Concern (Lai et al., 2020; Patel and Jernigan., 2020). At present, it has been found that the infection spread of severe acute SARS-CoV-2 is mainly via respiratory droplets and contact transmission from person to person (Lai et al., 2020; Li et al., 2020; Novel Coronavirus Pneumonia Emergency Response Epidemiology Team, 2020). The Chinese government has taken a series of effective administrative measures to interrupt the spread of the epidemic (Lau et al., 2020; Wu and McGoogan, 2020), including early diagnosis, patient isolation, symptomatic monitoring of contacts with suspected and confirmed cases, social distancing and community-based isolation, which played a pivotal role in limiting the COVID-19 outbreak (Wilder-Smith and Freedman, 2020; World Health Organization, 2020).

The hospital is not only the primary battlefield for pandemic prevention and treatment but also a high-risk place for epidemic transmission. During the COVID-19 pandemic, the risk of cross-infection in the hospital can be minimized by reducing hospital visits and exposure opportunities (Currie et al., 2016), strategies that have been widely adopted during the pandemic of various infectious diseases (Currie et al., 2016). Due to the pandemic, numerous non-emergency outpatient departments in Chinese hospitals were closed, causing most offline clinics to be unavailable to the public. While this crisis has presented the medical care delivery system with unparalleled challenges, COVID-19 has catalyzed rapid use of information and communications technology (ICT), such as telemedicine to deliver healthcare at a distance, offers an affordable, effective, and attractive option. Telemedicine including counseling, supervision, training, and psychoeducation in response to the pandemic, has been promoted and scaled up to reduce the risk of transmission (Li et al., 2021). Within this umbrella of telemedicine falls telepharmacy, the provision of pharmacist care by registered pharmacists and pharmacies through the use of telecommunications to patients located at a distance (Alexander et al., 2017; Win, 2017). Telepharmacy operations and services may include, but are not limited to, drug review and monitoring, dispensing, sterile and nonsterile compounding verification, medication therapy management (MTM), patient assessment, patient counseling, clinical consultation, outcomes assessment, decision support, and drug information (Alexander et al., 2017). Others are diagnostic and disease prevention services, therapeutic drug monitoring, and assessment of clinical outcomes (Hedima and Okoro, 2021).

During the pandemic, masses of patients in COVID-19-designated hospitals need therapy guidance from a pharmacist (Baldoni et al., 2019), and home-quarantined patients with chronic diseases requiring the long-term use of medications also need consultation from professionals (Li et al., 2021). Helping them manage home medicines is a problem that pharmacists urgently need to discuss and solve (Li et al., 2021). The current pharmaceutical care crisis and the need for social distancing have necessitated the need to adopt new initiatives for the treatment of patients (Anthony Jnr., 2021). Telepharmacy is expected to deliver timely pharmaceutical care while minimizing exposure to protect medical practitioners and patients and has been suggested as a method to maintain a continuum of pharmaceutical care for patients (Jnr, 2020).

As the world’s first country to respond quickly to COVID-19, the Beijing Pharmacists Association (BPA) screened 27 pharmacist volunteers with rich clinical experience and medication therapy management (MTM) qualifications from tertiary hospitals quickly establish an online voluntary pharmacy service team of “Cloud Pharmacy Care” (Li et al., 2021). To reduce the burden on the front-line medical teams, out of necessity, “Cloud Pharmacy Care” remote pharmacy service provides an interpretation of treatment plans from the medical teams and solutions for medication-related issues for patients and the public (Li et al., 2021). The BPA used Delphi’s method to develop an innovative remote pharmaceutical care framework in response to the current needs of epidemic prevention and control. Different from the telepharmacy provided by a traditional pharmacy, clinic, or medical institution, such as support to clinical services, remote education and handling of “special pharmacies” on medication dispensing safety, and prescription and reconciliation of drug therapies (Baldoni et al., 2019; Le et al., 2020; Pathak et al., 2020; Zhou et al., 2020), this study describes and analyses the framework, the results, and effects of internet-based remote pharmacy services supported by multidisciplinary professional pharmacists with MTM certification quickly response to the COVID-19 epidemic, with social software on smartphones for patients’ medication management and remote education. The innovative telepharmacy service aiming to provide a reference for international pharmacists.

## Methods

In response to the lack of medical resources in the epidemic area and the special situation of some home-quarantined patients requiring pharmacist guidance, in February 2020, the BPA quickly formulated an emergency remote pharmacy service framework through the Delphi method. The “Beijing Pharmacists Association” official account and medication consultation platform were built on the social software “WeChat” app. Patients can fill in personal information and health records to help pharmacists understand their overall health status. The service platform ensures patient privacy and information security.

### Establish a Remote Pharmacy Service Model

The BPA screened 27 pharmacists with rich clinical experience and MTM qualifications who work in the tertiary hospitals of the Beijing and Tianjin region quickly establish the voluntary team of “Cloud Pharmacy Care”, providing online free medication consultations. The professional major of pharmacists covers western medicine and traditional Chinese medicine, including cardiovascular, endocrinology, respiratory, gastroenterology, neurology, psychiatry, obstetrics and gynecology, and other specialties.

### Consultation Procedure

Patients can consult the individual pharmacist or the pharmacist team about medication-related issues in the form of texts, pictures, or voice messages from 8 a.m. to 8 p.m. each day. The content of the consultation includes treatment, medication reconciliation, chronic disease MTM, lifestyle guidance, and psychological counseling. The scope of services does not involve diagnosis, medical insurance reimbursement, medical expenses, or referrals.

### Implementation and Promotion of the Platform

After the service plan of the “Cloud Pharmacy Care” was approved by the Fangcang shelter hospitals, “A Letter to Fangcang Patients” was sent to the current patients by the pharmacist there. After the Fangcang shelter hospitals closed sequentially, the BPA issued service introductions to designated hospitals receiving COVID-19 patients, community health centers, and retail pharmacies in Beijing. Some medical institutions posted these service introductions in the outpatient pharmacy of the hospital for advertising.

### Service Quality Control

All licensed pharmacists received standardized and unified training before starting their consultations and have learned about the updated findings regarding COVID-19 in real-time. The pharmacists needed to fill in the consultation records of each patient, and the senior pharmacists (LD and JDC) reviewed the consultation questions and answers. For complicated problems, pharmacists discussed these issues with the other pharmacists to ensure the correctness of the answers. All the questions were analyzed descriptively, and the chi-square test was used for classification statistics and comparative analysis. The consulting system prompts those receiving consultations to rate the satisfaction of the service. The number of stars (1–5) represents satisfaction from low to high. A rating above four stars indicates a favorable experience.

## Results

### Establishment of the “Cloud Pharmacy Care” Remote Pharmacy Service

**FIGURE 1:**
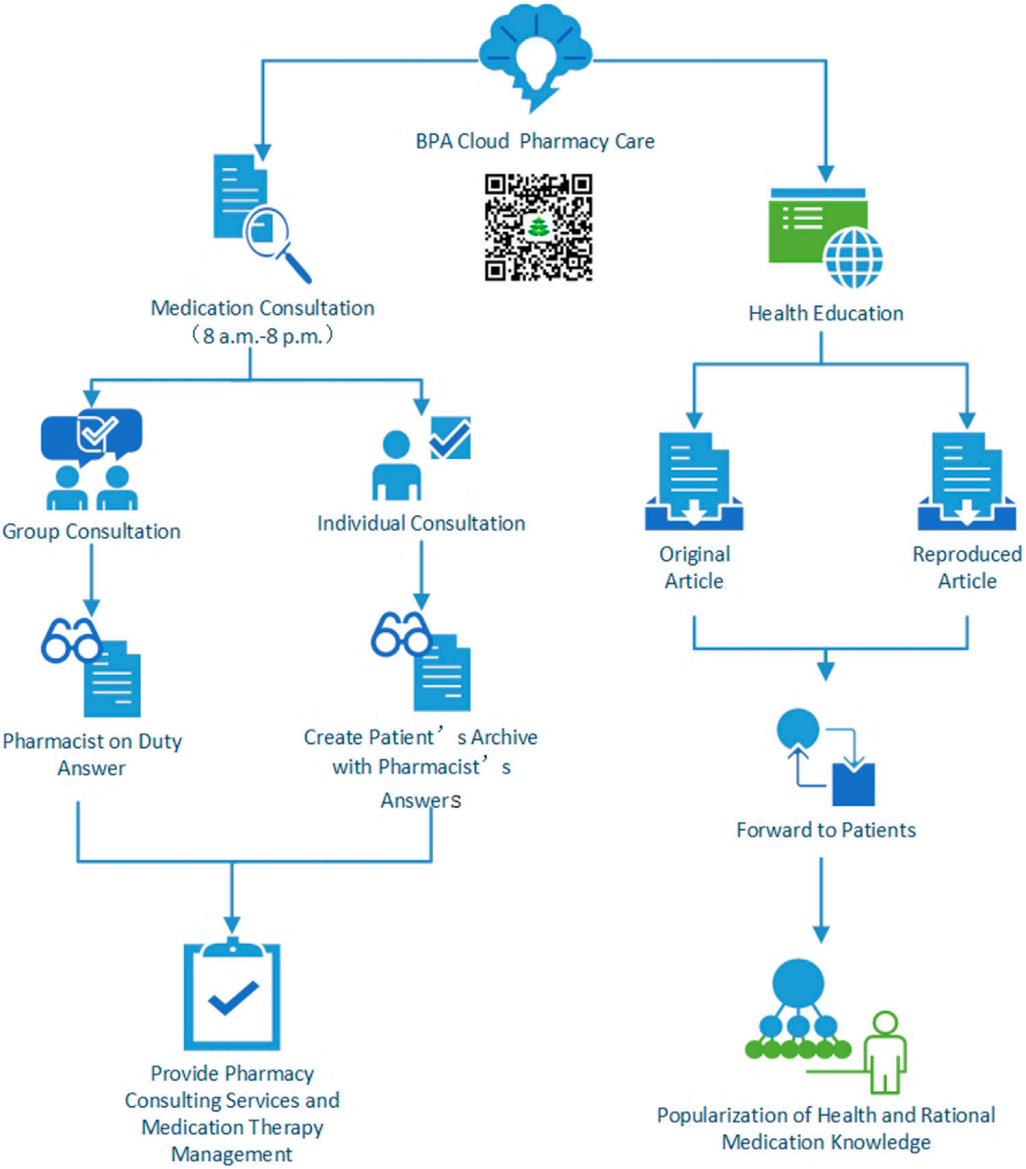
The “Cloud Pharmacy Care” pharmacy service mode.

After patients log in WeChat app and follow the “Beijing Pharmacist Association” official account, they then click “I need consultation” in the “Cloud Pharmacy Care” section of the public account interface to start a consultation. The “Cloud Pharmacy Care” pharmacy service framework is shown in Figure 1.

### The Work Mode of Medication Consultation

After receiving the patient’s consulting request, the on-duty pharmacist selects “I will answer” or recommends another specialized pharmacist on the team to answer. To protect patient privacy, patient information and consultation records during the consultation process can only be seen by the patient and the responding pharmacist. Patients can also choose to follow pharmacists according to their specialty and initiate a one-on-one consultation with the pharmacist. All consultations need to be completed within 4 h of an online request.

### Patient Management

Each pharmacist has a dedicated homepage, which can display personal basic information, time of visit, patient impression, patient education articles, answered consultations, and other information, from which patients can learn more about the pharmacist’s information. The pharmacist’s homepage also displays a QR code unique to the pharmacist. The patient can follow the pharmacist by scanning the QR code, which is convenient for secondary transmissions. Pharmacists can manage patients who follow them, view their health information, add diagnosis and treatment files to patients’ records, and add tags in the settings.

### Patient Education

Promoting the knowledge of rational drug use to the public is another important task of the “Cloud Pharmacy Care” service, and this is consistent with the push function of the WeChat platform. Pharmacists can compose original messages or reprint popular science articles, graphic illustrations, animations, or videos. Patient education materials are published and can be sent directly to patients to facilitate education regarding rational medication use.

### Tele-Consultation Results

#### Consulting Object

From February 28 to April 27, 2020, during the worst period of the epidemic in China, “Cloud Pharmacy Care” had 1,432 views, 66 followers, and received 39 counseling cases in the two previous months. The basic characteristics of the consultant are shown in Table 1. The average age of the patients was 42 years old, where the youngest was 2 years and 7 months old, and the oldest was 85 years old. Counseling was available for patients of all ages; 82.05% were young and middle-aged. Four people requested consultations on behalf of others, among which 2 were adults seeking consultations for their parents, and two were parents seeking consultations for minors.

**TABLE 1:** The basic characteristics of the consultant.

		Number of consultations	No. (%)
Gender			
	Male	15	38
	Female	24	62
**Age**	Years		
	<18	3	8
	18–40	18	46
	41–65	14	36
	>65	4	10
**Consultant**			
	Patient	33	85
	Replacer	4	10
	Medical personnel	2	5

### Consulting Content

**FIGURE 2:**
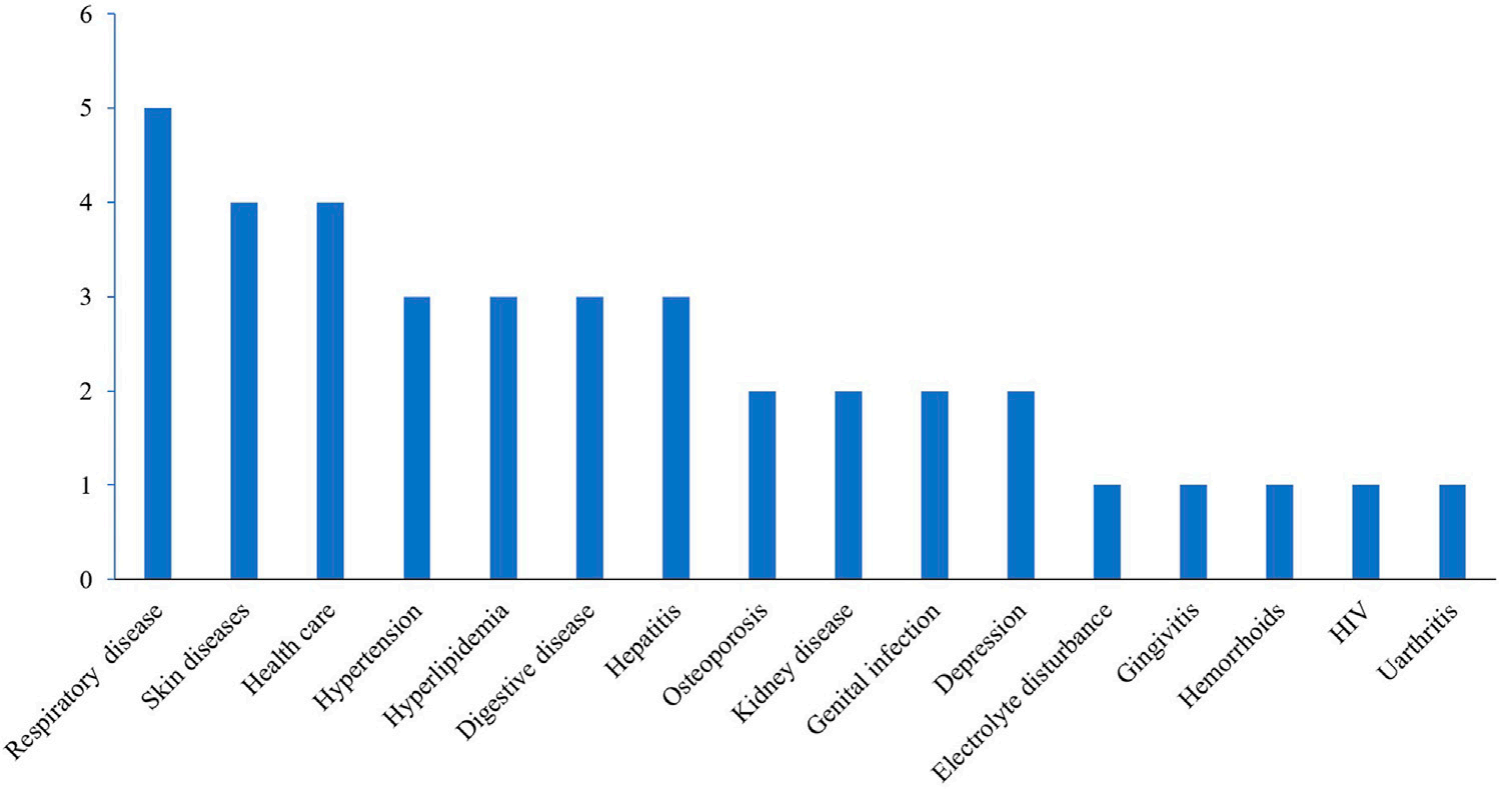
Disease distribution involved in the consultation content.

**FIGURE 3:**
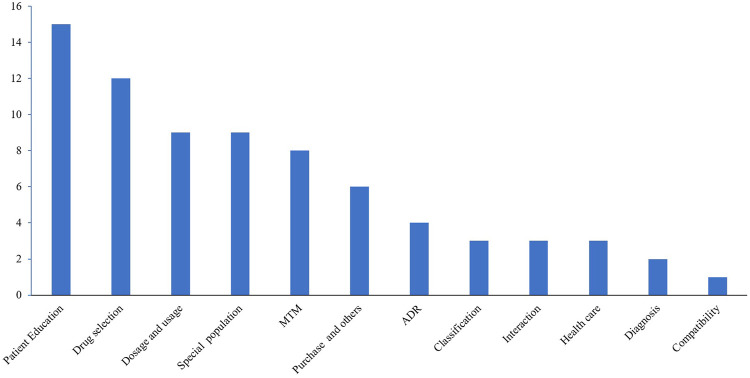
The classification distribution involved in the consultation content.

In a single consultation, there were a maximum of 13 items and a minimum of two items. On average, each consultant needed to answer four to five items. In the consultations, 35 cases (89.7%) were related to the use of medicines or health products, and 4 cases (10.3%) involved disease management and the use of supplements. The diseases and classification distribution involved in the consultation content are shown in Figure 2 and Figure 3. The chi-square test showed that the classification of consultation questions does not correlate with age and gender. Three representative examples of

The pharmacist’s solution and analysis of patients’ medication problems were given in Table 2.

**TABLE 2:** Representative examples of the consultations on “Cloud Pharmacy Care".

Content	Case 1	Case 2	Case 3
**Chief complain**	Stomach ache that affected sleep, anxiety	Swelling of the left foot and ankle	Eyelid edema
**Subjective/objective**	Male, 39 years old	Female, 60 years old	Male, 2 years and 7 months
	**Gastric ulcers**: stomachache affected sleep, gastroscopy could not be done during the epidemic, need painkillers to improve the sleep	**Hypertension:** blood pressure approximately 140/80 mmHg	Eyelid edema for 2 days, the 3rd times onset in the last month
	**Anxiety:** Patient experiencing recurring symptoms	**Gout:** sudden swelling of the left foot and ankle recently, consider gout recurrence	Urine protein 4+, urine occult blood 2+, urine red blood cells 660.80/μl, urine white blood cells 29.4/μl
	**HIV:** take medications regularly		
**Past medical history**	Anxiety (was on escitalopram 10 mg QD)	Gout (was on colchicine)	None
**Current Medications**	Atripla (tenofovir + lamivudine + efavirenz) 1 tablet p.o. QD; omeprazole capsule 20 mg p.o. QD; ibuprofen sustained-release 0.3 g p.o. BID	Indapamide 2.5 mg p.o. QD; aspirin 100 mg p.oQD.	Loratadine 5 mg p.o. QD
**Drug therapy problems**	**Gastric ulcers:** effectiveness-frequency inappropriate	**Hypertension:** safety-undesirable effect; effectiveness-more effective drug available	Eyelid edema: indication-untreated condition, need a definite diagnosis
	**Anxiety:** indication-untreated condition	**Gout:** safety-undesirable effect, indication-untreated condition	Loratadine: effectiveness-not effective for the condition
	**HIV:** safety-undesirable effect		
**Assessment**	**Gastric ulcers:** omeprazole dosage too low, and not recommended to take NSAIDs	**Hypertension:** adverse drug reaction, hyperuricemia may be associated with indapamide; blood pressure poorly controlled, need different drug product	Suspected to be nephritis or nephrotic syndrome
	**Anxiety:** needs additional drug therapy	**Gout:** needs additional drug therapy	
	**HIV:** adverse drug reaction, Efavirenz may cause drug-induced anxiety		
**Plan**	Taking omeprazole 20 mg p.o. BID, withdrawal ibuprofen	Start amlodipine 5 mg p.o. QD, aspirin 100 mg p.o. QD, colchicine 1 mg p.o. QD	Recommended to go to the hospital
	Re-start escitalopram 10 mg p.o. QD		
**Follow-up**	Stomachache relieved 1 week later, and the anxiety state was well controlled 1 month later	Gout relieved, blood pressure approximately 130/80 mmHg 1 month later	Diagnosed with “nephrotic syndrome”, treatment with methylprednisolone 10 mg tid, 3 days later

### Service Quality Evaluation

All consultations were completed within 4 h, and the completion rate was 100%.

All those receiving consultations rated the teleconsultation service of the pharmacists: 38 people gave a rating of five stars and one person rated the consultation three stars, yielding a positive rate of 97.4%. There were no complaints from consultants, and no doctor-patient disputes occurred. The patients generally commented that the pharmacist responded in a timely, earnest, professional, patient-caring and enthusiastic manner, e.g., “During the epidemic, the guidance of the pharmacist is very valuable”, “Thank you for this lovely platform.”

For acute and severe patients, the pharmacists would follow up on the patient’s illness and medication status after the consultation. Examples of follow-up records are shown in Table 2. The senior pharmacists commented on the records of all questions and answers, and these comments were all scientific and appropriate. The telepharmacy service during the epidemic is an emerging service. We are still exploring the working framework and have been constantly improving the process of plan, do, check and act (PDCA).

## Discussion

Disasters and pandemics pose unique challenges to healthcare delivery. The current COVID-19 pandemic reminds us of the importance of using telemedicine to deliver care, especially in reducing the risk of cross-contamination caused by close contact during infectious public health emergency responses (Feng et al., 2020; Hollander and Carr, 2020; Smith et al., 2020; Zhou et al., 2020). Although innovative telepharmacy services will not solve all patient needs, they are well suited for scenarios in which infrastructure remains intact and pharmacists are available to reduce the public panic and address the patients’ medication problems as a supplement to a remote assessment in primary care (Greenhalgh et al., 2020; Ibrahim et al., 2020) and mental health services (Yang et al., 2020; Zhou et al., 2020). In addition to the “Online Pharmaceutical Monitoring” service launched by the Tongji Medical College of Huazhong University of Science and Technology for the patients in the Fangcang shelter hospital, “Cloud Pharmacy Care” is the first online pharmacy consulting service platform organized by a professional society and composed of MTM-qualified pharmacists in China, providing free pharmaceutical care for the public during the COVID-19 pandemic (Li et al., 2021).

### Construction of Telepharmacy Services

Telepharmacy models have been implemented in various countries of the world despite variations in healthcare systems. In China, “Mobile Internet + Medical Care” oriented by the big data value chain is gradually changing medical practices and processes (Wang et al., 2017). During the outbreak of the COVID-19 pandemic, the long-term medication-use problems of patients with chronic disease were effectively solved by “Cloud Pharmacy Care”, such as respiratory disease, skin diseases, hypertension, hyperlipidemia, osteoporosis, etc. The top five drug-related issues include the choice of medications, the dosage and usage of drugs, medications for special populations, MTM of chronic diseases, and adverse drug reactions. In particular, through remote consultation by the pharmacists, a patient’s disease could be discovered and diagnosed in time (such as the child’s parents mistakenly thinking the nephrotic syndrome was allergic edema). If discomfort or adverse drug reactions occurred (such as increased uric acid caused by indapamide), treatment was adjusted promptly.

Telepharmacy can alleviate the queuing problem and reduce the cost of transportation for patients. One of the advantages is that healthcare professionals can efficiently monitor patients’ indicators and provide suggestions at any time. This is a low-cost, convenient and stable pharmacy service model that is not only suitable for emergencies but can also extend to unhealthy people, patients with chronic diseases, and healthy people who need pharmacy consultation in daily life. However, due to the short establishment time and insufficient publicity, the public’s attention and the number of consultations was not very high in the first 2 months of the establishment of “Cloud Pharmacy Care”. In non-pandemic times, this type of telepharmacy can facilitate patient communication when patients have barriers such as living in a rural/remote location, mobility challenges, poor access to transportation, or inflexible work or caregiving schedules.

### Advantages and Value Based on Social Software

Traditional ways of communication are inefficient and painfully slow. Healthcare providers are currently experiencing transformational change and turning to social media to network, connect, engage, educate, and learn (Toney et al., 2015; Benetoli et al., 2017). As an instant messaging tool, the multifunctional WeChat official account consulting platform has many natural advantages. First, as the most widely used social software in China with nearly 1.2 billion active users, the operation is simple and not subject to excessive restrictions on age and cognitive behavior, providing a convenient method for medication consultation. Second, the “Cloud Pharmacy Care” medication consultation platform has multiple interactive forms available, such as text, voice, picture, and video. Patients can upload medical records, test and examination results, and all consultation records can be archived. At the same time, pharmacists can edit the patient’s file, which is convenient for pharmacists to classify and manage patients. Third, WeChat’s exclusive account login mode protects the privacy of patients, and patients can confidentially consult with pharmacists about personalized medication use. Last but not the least, from the perspective of technical implementation, with the advent of COVID-19, remote pharmacy care based on WeChat is easier to realize and apply than to establish a new APP.

### Risk Management

Telemedicine adoption requires a whole-system strategy. Embedding telemedicine into routine service delivery by all healthcare providers is the most effective way of ensuring telemedicine can be readily used during emergencies, and it requires operational networks, telemedicine policies and procedures, and technology infrastructure that can be scaled up during times of epidemic. Accordingly, there are social, organizational, and technological factors that impact the widespread adoption of telepharmacy platforms by patients and medical practitioners (Bokolo, 2021). Among others, ethical considerations must be taken into account (Shokri and Lighthall, 2020). Telepharmacy services require professional knowledge, communication skills, empathy, and medical risk awareness of pharmacists. In 2018, the Chinese Pharmacist Association, Chinese Pharmaceutical Association Hospital Pharmacy Professional Committee, and four other associations jointly issued the “Expert Consensus for Pharmacists to Provide Internet Science Popularization and Consulting Services”, encouraging pharmacists to actively explore new models of Internet pharmacy services with a rigorous and scientific attitude (Chinese Pharmacists Association, Chinese Pharmaceutical Association Hospital Pharmacy Professional Committee et al., 2020). This expert consensus highlights that pharmacists’ professional activities on the internet platform must follow basic principles, ethics, and professional responsibility standards and include conducting risk management (Chinese Pharmacists Association, Chinese Pharmaceutical Association Hospital Pharmacy Professional Committee et al., 2020). In the Spanish Society of Hospital Pharmacy position statement (Morillo-Verdugo et al., 2020), the ASHP statement on telepharmacy (Alexander et al., 2017), and practice advancement initiative 2030 (ASHP Practice Advancement Initiative, 2020), the guidances also recommend that pharmacists use health information technologies to advance their role in patient care and population health.

The limitation of this paper is that the sample size of free online pharmacy consultation provided in this paper is small, because it only happened in the 2 months when the COVID-19 pandemic was the most severe in China, and also the 2 months when people in the whole country were quarantined at home. After that, the Chinese people resumed their normal life and were free to go out to the hospital. However, the framework of online pharmaceutical consultation has been successfully established, and then gradually transferred to paid pharmaceutical care.

## Conclusion

Under the COVID-19 pandemic, a remote pharmacy service platform “Cloud Pharmacy Care” based on social software was quickly constructed and applied to help patients solve medication-related problems. In general, patients of all ages use the telepharmacy service for both acute and chronic conditions under the COVID-19 epidemic. Most patients asked questions related to their health or medications, and females preferred online consultation. Patients were more likely to ask questions related to prescription and OTC medications than questions about botanical and dietary supplements, and they are primarily interested in drug efficacy, adverse effects, and chronic disease management. Through telepharmacy services, the patients can keep in touch with their pharmacists. The study implies that the timely and interactive consultation platform helps to carry out medication management for chronically ill patients, improve patients’ medication adherence, and improve medical quality, and it can play a positive role in promoting the popularization of safe medication knowledge. Findings from this study highlight that telepharmacy is important and can be adapted to support the treatment of patients during and after the pandemic. Because the COVID-19 epidemic was effectively controlled in China, the free remote pharmaceutical care was carried out for a short time, and the sample size recorded was small. However, the remote pharmacy service framework we established is scientific and feasible. In non-pandemic times, this type of telepharmacy can facilitate patient communication. Future research should be directed at increasing the accuracy of collected patient information, documenting pharmacists’ interventions, and measuring patient outcomes using an interactive model through the telepharmacy service.

## Data Availability

The raw data supporting the conclusions of this article will be made available by the authors, without undue reservation, to any qualified researcher.
